# 2D Slab Models of Nanotubes Based on Tetragonal TiO_2_ Structures: Validation over a Diameter Range

**DOI:** 10.3390/nano11081925

**Published:** 2021-07-26

**Authors:** Oleg Lisovski, Sergei Piskunov, Dmitry Bocharov, Stephane Kenmoe

**Affiliations:** 1 Institute of Solid State Physics, University of Latvia, LV-1063 Riga, Latvia; oleg.lisovski@gmail.com (O.L.); Dmitrijs.Bocarovs@cfi.lu.lv (D.B.); 2 Transport and Telecommunication Institute, LV-1019 Riga, Latvia; 3 Department of Theoretical Chemistry, University of Duisburg-Essen, D-45141 Essen, Germany; stephane.kenmoe@uni-due.de

**Keywords:** nanotubes, TiO_2_, water adsorption, slab model, DFT, water splitting

## Abstract

One-dimensional nanomaterials receive much attention thanks to their advantageous properties compared to simple, bulk materials. A particular application of 1D nanomaterials is photocatalytic hydrogen generation from water. Such materials are studied not only experimentally, but also computationally. The bottleneck in computations is insufficient computational power to access realistic systems, especially with water or another adsorbed species, using computationally expensive methods, such as ab initio MD. Still, such calculations are necessary for an in-depth understanding of many processes, while the available approximations and simplifications are either not precise or system-dependent. Two-dimensional models as an approximation for TiO_2_ nanotubes with (101) and (001) structures were proposed by our group for the first time in Comput. Condens. Matter journal in 2018. They were developed at the inexpensive DFT theory level. The principle was to adopt lattice constants from an NT with a specific diameter and keep them fixed in the 2D model optimization, with geometry modifications for one of the models. Our previous work was limited to studying one configuration of a nanotube per 2D model. In this article one of the models was chosen and tested for four different configurations of TiO_2_ nanotubes: (101) (*n*,0), (101) (0,*n*), (001) (*n*,0), and (001) (0,*n*). All of them are 6-layered and have rectangular unit cells of tetragonal anatase form. Results of the current study show that the proposed 2D model is indeed universally applicable for different nanotube configurations so that it can be useful in facilitating computationally costly calculations of large systems with adsorbates.

## 1. Introduction

Performing quantum-chemical calculations of large systems, e.g., nanotubes (NTs) of realistic diameters with some chemical molecule species (e.g., water) adsorbed on one or both wall sides with computationally-costly methods such as ab initio Molecular Dynamics is one of the current problems in computational chemistry. The systems which modern supercomputers are able to simulate within a reasonable time are usually too small to match the finest systems accessible in experiments. Therefore, some computational shortcuts were developed to bridge this gap. Among others, there is an established approach to use 2D structures as an approximation to 1D NTs.

Other researchers have offered shortcuts for simulation of mechanical properties [1,2] and electronic properties [3,4] of NTs with a non-periodic cluster model constructed of an NT segment. The drawback of the method is unphysical effects on the model boundaries which results in inaccuracies of electronic structure reproduction. There is also another, zone-folding approach [5,6,7], which allows thermodynamic NT properties. This approach benefits from exploiting model periodicity but is not capable of taking NT curvature into account. Hence, there is still a necessity for a 2D-periodic model able to describe the electronic properties of NTs.

Previously, authors of the present paper have proposed two working models of water adsorption simulation on a surface of a TiO_2_ nanotube via constrained slabs [8]. This included a so-called Fixed Volume Slab (FVS) model which was based on slab lattice parameter modification with respect to NT constants, and a so-called Constrained Fixed Volume Slab (CFVS) model which is based not on a regular TiO_2_ slab with modified lattice constants, but on a 2D structure with internal coordinates based on nanotubular unit cell geometry, and with keeping coordinates of one of the TiO_2_ units fixed. The model construction was performed at the DFT level for further use at the ab initio MD level. More details are available in [8].

The aforementioned models have already been successfully used for their purpose, ab initio MD simulations of water adsorption on the surface of TiO_2_ NTs. This included a study of water adsorption on 2D-representation of 9-layered TiO_2_ nanotube, on clean and doped surfaces [9]. The CFVS model was also used to study the structure, energetics, and the photoelectrochemical oxidation of water on the aforementioned structures by means of the DFT + *U* method and a plane-wave-based computational code Quantum Espresso [10,11]. The results of these studies are summarized in a review [12].

Further on, the 2D slab models were validated over a diameter range: FVS—against (101) 6-layered (0,*n*) NTs (adsorption on the inner surface) with *n* ranging from 8 to 50 and diameter—from 2.85 to 16.50 nm, and CFVS—against (001) 9-layered (*n*,0) NTs with *n* in the range from 8 to 50 and diameter—from 1.9 to 6.32 nm. Such breadth allowed to cover all necessary domains: small NTs with prominent curvature effects, medium NTs, and large NTs with properties converging to a regular TiO_2_ slab. Configuration of an NT wall made each of the models more suitable for a specific NT type, please refer to our previous work for a detailed explanation [8,13].

The next step of 2D model development, considered in the present article, is dedicated to validating a single slab model against different configurations of TiO_2_ NTs. In the previous work, each of the 2D models was validated against a diameter range of one specific NT configuration. In turn, validation of a single 2D model against different NT configurations would create a more solid fundament for concluding on the model’s universality. Since the FVS model is less time-consuming in terms of construction, in the present study it was decided to limit the scope to the FVS model and TiO_2_ NTs of four different configurations: (101) 6-layered (*n*,0) and (0,*n*), and (001) 6-layered (*n*,0) and (0,*n*), with *n* ranging from 12 to 40 for each NT with an increment of 4. In every case, only adsorption on the outer surface is studied since it makes the most prominent contribution to the water adsorption process. The validation criteria are single water molecule adsorption energy and band gap maximum/minimum positions calculated for structures without water adsorbed.

Obviously, water adsorption causes differences in band edge positions. Still, it is not the task of the current paper to study the effects of water adsorption, since more advanced approaches and models are needed for this. Instead, here the comparison is performed between the full-size NTs and their 2D models, and the most important is to consistently compare equivalent parameters, i.e., between two systems with water, or between two systems without water.

The general motivation for using NTs and TiO_2_ as the material of the study is as follows.

Among many functional nanomaterials, nanostructured TiO_2_ are of great interest due to their unique properties and numerous practical applications [14,15,16,17]. The main interest in titania-based nanomaterials is basically associated with such high-efficient applications as lithium-ion batteries, solar cells, gas sensors, supercapacitors, etc., [18,19,20,21,22,23]. Moreover, active investigations are related to the photocatalytic activity of titania-based materials, including nanopowders and thin films [24,25,26,27,28,29]. Due to chemical stability, non-toxicity, low cost, and high availability, titanium dioxide is considered the most promising photocatalyst for the degradation of organic pollutants in water and air, as well as for water splitting and hydrogen production [30,31,32]. The additional importance of titania is related to its applications as protective coatings in microelectronic and optical devices and as luminescent compounds [33,34,35,36,37,38].

Titanium dioxide was the pioneering material in the field of photocatalytic water splitting for hydrogen generation. For this purpose, it was used for the first time in 1972 by Honda and Fujishima [39], and in the following 5 decades, a lot of attention was attracted to this material due to its prospective characteristics, such as its plenitude, low costs, non-toxicity, efficient separation and migration of charges. In addition, the position of the valence band maximum (VBM) is well-matching to hydroxyl group oxidation potential, thus providing suitable conditions for this half-reaction. On the other hand, the bandgap of TiO_2_ is wide (~3.2 eV in the form of anatase), and, consequently, the position of the conduction band minimum (CBM) is too low with respect to hydrogen cation reduction potential [40]. For efficient photocatalysis, gaps in the range of 1.5–2.5 eV are usually mentioned as optimal, hence a necessity to modify the material arises.

One of the ways of improving photocatalyst efficiency is the reduction of its dimensionality, to 2D and further on to 1D materials, since lower dimensionality provides shorter distances of charge migration and more efficient charge separation. The photocatalytic potential of nanotubes, nanorods, and nanowires is frequently studied. The favorable effect of low dimensions is especially prominent in NTs due to their hollow structure [41,42].

A particular problem of lowering dimensionality of TiO_2_ structures is the increase in the bandgap. One of the common ways to overcome this issue is to use dopant atoms in order to narrow the bandgap. This was a particular subject of interest for our group, and a series of studies have been completed. The last publication of this series [43] resulted in recommendations on doping TiO_2_ NTs in order to narrow the bandgap and align valence band maximum and conduction band minimum with respect to corresponding redox potentials. Carbon, nitrogen, sulphur and iron atom defects were studied, and the study concluded with a recommendation to combine S and N defects for the optimal band edge alignment.

While NTs are successfully fabricated from various materials, including titania [41], their computational studies are not abundant, mostly because the simulation of realistic NTs and other 1D nanostructures is too costly. The diameter of the finest synthesized TiO_2_ NTs is on the order of 10 nm, and it implies thousands of atoms in a computational model. This is still accessible by some computational codes, such as CRYSTAL [44], when it is possible to use rototranslational symmetry in order to cut the required computational resources. Thus, when there are some chemical species in the disordered phase adsorbed on such a structure, symmetry exploitation becomes inapplicable, and other solutions are needed. This closes the loop of discussion back to the motivation of developing 2D slab models of nanotubes.

## 2. Computational Details

Two-dimensional FVS models of the aforementioned four configurations of TiO_2_ NTs were developed at the DFT theory level, using hybrid exchange functional B3LYP and LCAO basis sets with localized Gaussian-type functions. The periodic code CRYSTAL [44] developed by Italian scientists at Torino university was used. In the program, there is a possibility to use rototranslational symmetry to essentially decrease needed computational resources.

In brief, the B3LYP functional was used with altered non-local HF exchange contribution (14% instead of the default 20%). The following basis sets were used: for Ti—efficient core potential basis set developed by Hay and Wadt [45] with 411sp−311d configuration for the outer shell, for O—a full-electron basis set with 6s−311sp−1d configuration [46], for H—a TZVP basis set [47]. The reasoning consisted in finding a tandem of functional and basis sets that ensure the best match of VBM and CBM to the experimental data. For the explicit procedure of basis set and exchange-correlation functional choice, the reader is kindly referred to our previous work [43].

**Figure 1:**
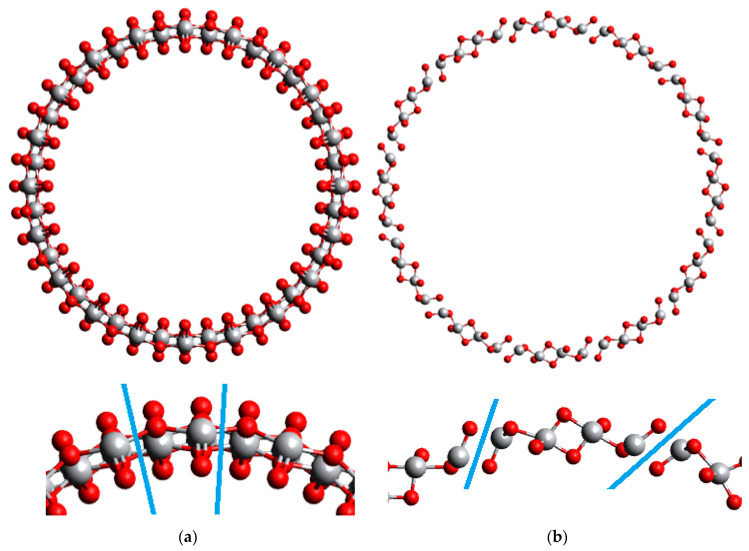
Structures of 6-layered (101) TiO_2_ NTs. Unit cells are depicted with blue lines in the magnified segments. (**a**) (*n*,0) configuration; (**b**) (0,*n*) configuration.

**Figure 2:**
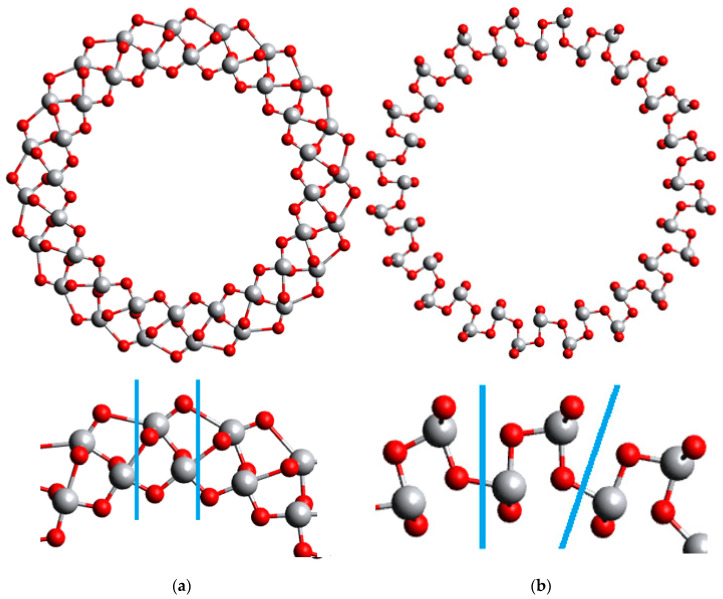
Structures of 6-layered (001) TiO_2_ NTs. Unit cells are depicted with blue lines in the magnified segments. (**a**) (*n*,0) configuration; (**b**) (0,*n*) configuration.

In our previous work [8,13], the 6-layered (101) NTs and 9-layered (001) NTs had a prominent difference in the wall thickness and, consequently, radial distances until every non-equivalent atom. In the present study, both (101) and (001) NTs are 6-layered, which makes their wall thickness more similar, although the (101) configuration is thinner, see Figure 1 and Figure 2 below. In Table 1 and Table 2 main structure parameters of the (101) NTs with (*n*,0) and (0,*n*) chirality indices are presented, and in Table 3 and Table 4—parameters of the (001) NTs, also with (*n*,0) and (0,*n*) chirality indices. The chirality index is defined as the number of elementary units replicated circumferentially in an NT-ringed unit. When there is water in the model, it implies coverage of one water molecule per unit cell. The same will be true for 2D models with water.

In Table 1 it is visible that for pristine NTs *a* increases along with diameter growth, with approximately the same amplitude as in the first case. The *a* × *b* product also changes while *b* decreases. The magnitude of change is essentially higher for the *b* constant. In the case of NTs with water adsorbed, the change in *a* constant is even less prominent, while *b* changes within approximately the same range both for the case with water and without. Similar considerations are applicable also to (0,*n*) configuration. The largest difference is in larger NT diameters due to the rectangular unit cell rotated by 90°, another difference is the interchanged *a* and *b* constants.

When comparing Table 1 and Table 2 to Table 3 and Table 4, it is visible that the general trends are similar. The difference is that in the case of (001) NTs, there is no essential difference in diameter between (*n*,0) and (0,*n*) configurations with identical *n*. Another important difference is that *a* × *b* product for (001) NTs is several times lower than for (101) NTs, but the magnitude for its change is higher.

**Figure 3:**
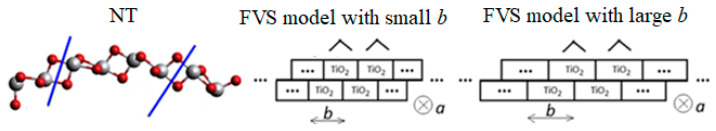
Basic illustration of the 2D FVS model principle.

In our previous studies [8,13] we dealt with two types of models—the aforementioned FVS and CFVS. In this study, we use only the FVS model (Figure 3) for multiple NT configurations, therefore we will limit ourselves to a brief illustration of its principle.

The lattice constants are taken from an NT and used in a TiO_2_ anatase slab instead of its original constants. It is important to follow the correct order of constants. So, in the (*n*,0) case *a* and *b* from an NT become *a* and *b* in the 2D model, while in the (0,*n*) case *a* and *b* from an NT become, in turn, *b* and *a* in the 2D model. The lattice parameters of the 2D model are fixed to the same value throughout geometry optimization. In the present study, water adsorption on the outer NT surface is investigated, so the *b* constant corresponding to the outer NT layer is used. There are also options to use lattice parameters from a reference NT with a clean surface, or with adsorbed water, or the average of these two options.

## 3. Results and Discussion

### 3.1. The (101) NTs with (n,0) and (0,n) Configuration

**Figure 4:**
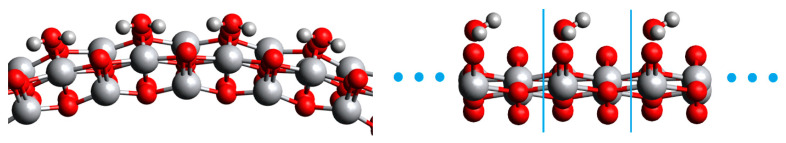
Fragment of a 6-layered (101) TiO_2_ NT (40,0) and its 2D FVS model, with a multiplied unit cell.

**Figure 5:**
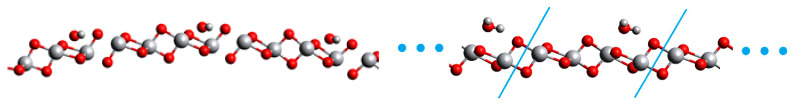
Fragment of a 6-layered (101) TiO_2_ NT (0,40) and its 2D FVS model, with a multiplied unit cell.

The results section for both (101) and (001) configurations begins with a discussion on full-size NT and 2D model geometries. In Figure 4 and Figure 5, geometries of reference NTs (40,0) and (0,40) are presented, with their respective 2D models. It is visible that the slab model looks similar to the original NT, although there is no curvature in the planar structure. In both cases, water adsorbs with oxygen pointing at surface titanium, but in the full-size NT models hydrogens point at two surface oxygens symmetrically, while in the 2D model one of the water hydrogens points somewhat outwards. Such a trend was in general observed for the rest diameters of NTs. Two-dimensional models of smaller nanotubes, with a chirality index *n* = 12–16 are more distorted, while this problem is alleviated starting from a chirality index of 20 and above. This is natural since curvature effects are prominent for smaller NTs, and in the 2D models there is no curvature accounted.

**Figure 6:**
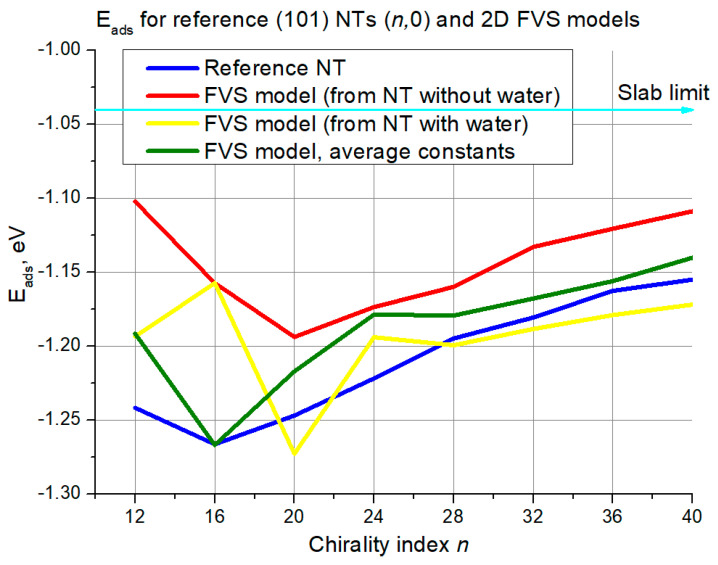
Adsorption energies for (101) NTs (*n*,0) and their 2D FVS models. The cyan line with an arrow denotes the slab limit for water adsorption energy.

Next, the first validation criterion is discussed—the water adsorption energy. One graph per NT configuration is included. Each graph contains water adsorption energies for a series of reference NTs, shown with dark blue curves, and energies for three variations of the FVS model—with constants adopted from a NT without water (red curve), with water (yellow curve), and with constants calculated as an average between these two (green curve). A straight cyan line is added to each graph to indicate the regular slab limit for water adsorption energy. In Figure 6 results for the (101) (*n*,0) structures are shown.

It is seen that in the left part of the graph there are essential fluctuations. The reference curve itself does not change monotonically as the chirality index grows, there is a minimum at *n* = 16, which was reproduced by the FVS model variation with averaged constants. Still, the performance of 2D models in the domain of low chirality index is less accurate than for larger diameters. The largest deviation is ~0.15 eV for the reference NT and FVS model based on NTs without water. For the rest variations and neighboring chirality index the deviation is mostly around 0.05–0.10 eV.

The chaotic behavior of 2D FVS model curves stops at *n* = 24. Since then, the growth of all curves is rather monotonical in the direction to the slab limit (−1.04 eV), and agreement with the reference curve becomes better. It is also observed that the FVS model based on NTs without water underestimates the adsorption energy in this interval, while the variation based on NTs with water—overestimates for all cases except for *n* = 24. The deviation is slightly less than 0.05 eV for the red curve, and several times lower for the yellow and green ones. The 2D FVS models with averaged constants provide average results between the two aforementioned cases, which is in good agreement with our previous study [12]. They also provide somewhat better agreement than the yellow curve towards larger NT diameters.

**Figure 7:**
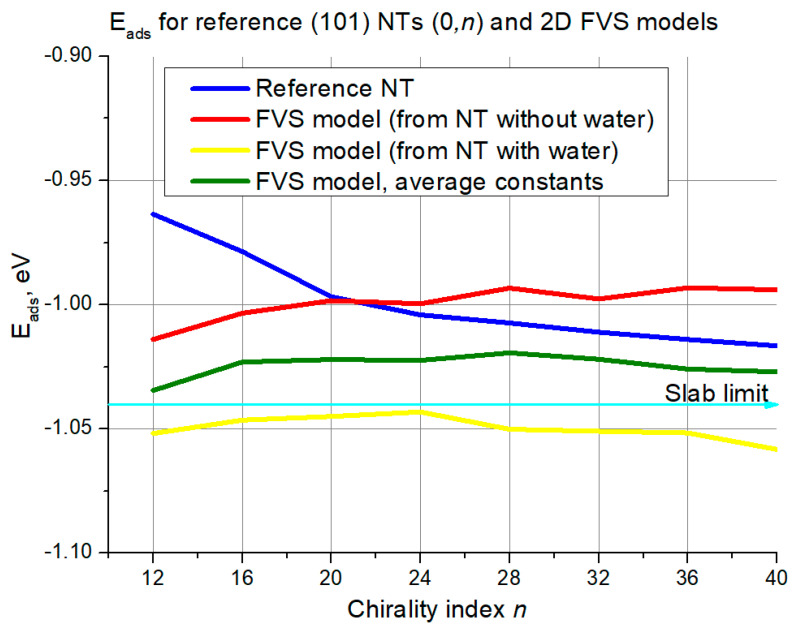
Adsorption energies for (101) NTs (0,*n*) and their 2D FVS models. The cyan line with an arrow denotes the slab limit for water adsorption energy.

The results section continues with Figure 7 and results for the (101) (0,*n*) structures.

In this case, there was no such chaotic curve behavior in the interval of small diameters for all the curves as in the case of (*n*,0) structures. Still, the deviation from the reference curve is again the highest for the smallest diameters, with the yellow curve being the least accurate with its ~0.08 eV deviation. Again, the curve of the FVS model with averaged constants is in between the two other models. For this curve, good agreement with the reference begins at *n* = 24–28, with an error of around 0.025 eV and below. The red curve crosses the reference at *n* = 20, but this agreement is not consistent since later on the red curve diverges from the blue one. The yellow curve continues towards larger chirality indices in parallel to the reference at around 0.04 eV distance. The reference curve and the curve of the FVS model with averaged constants both converge to the slab limit (−1.04 eV).

**Figure 8:**
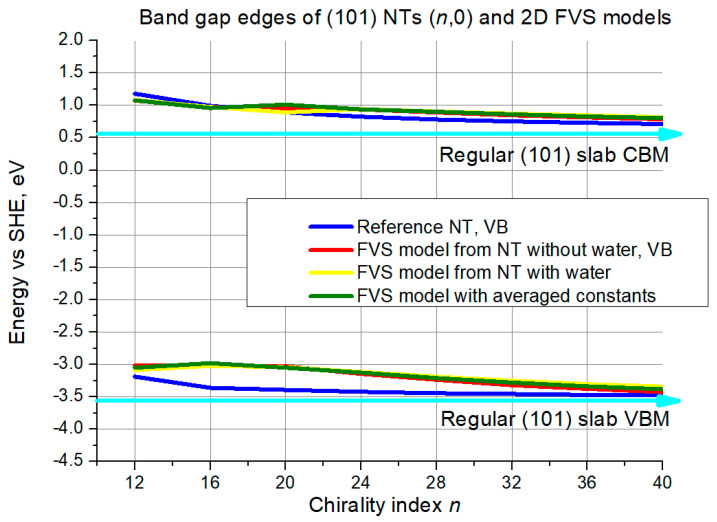
VB top and CB bottom edge positions for (101) NTs (*n*,0) and their 2D FVS models. The cyan lines with arrows denote slab limit for VB maximum and CB minimum.

**Figure 9:**
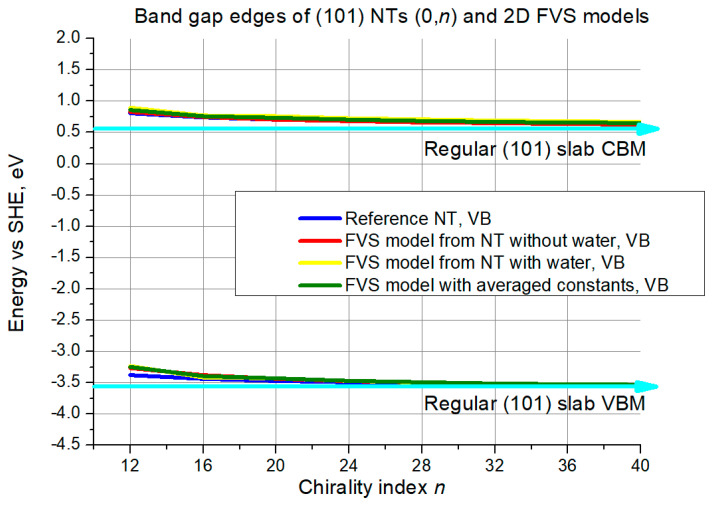
VB top and CB bottom edge positions for (101) NTs (0,*n*) and their 2D FVS models. The cyan lines with arrows denote slab limit for VB maximum and CB minimum.

Next, the criterion of band edge positions will be discussed based on illustrations in Figure 8 and Figure 9.

As is seen in Figure 8, in the slab limit the CBM is slightly above 0.5 eV level, and VBM—slightly below −3.5 eV, see the cyan lines with arrows. At *n* = 12, all curves including the reference are approximately 0.5 eV higher than the slab limit. For the CBM, the agreement between all the curves is very good and it is difficult to distinguish between them. Still, the curve for the 2D models with averaged constants is between the other two starting from *n* = 24 approximately. The red curve is the closest to the reference.

For the VBM, the reference curve approaches the slab limit much faster than the others. The curves for all the three FVS models gradually approach the reference, reducing the deviation from around 0.4 eV at *n* = 16 to almost 0 at *n* = 40. Again, the red curve is closest to the reference in the interval of the larger NTs, and the green curve is in the middle.

For the (101) (0,*n*) structures the situation is similar, with some minor differences. The curves also start above the slab level, this time with a somewhat lower difference of around 0.25–0.4 eV. All the curves converge to the slab limit in the same way, the convergence is more prominent for the VBM. Agreement between the different curves is very good.

### 3.2. The (001) NTs with (n,0) and (0,n) Configuration

**Figure 10:**
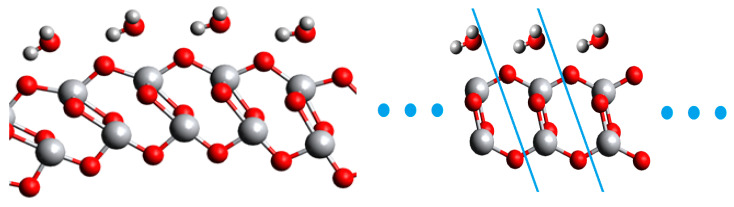
Fragment of a 6-layered (001) TiO_2_ NT (40,0) and its 2D FVS model, with a multiplied unit cell.

**Figure 11:**
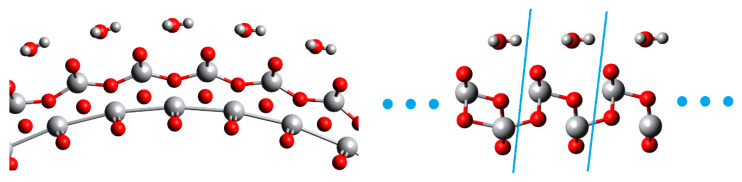
Fragment of a 6-layered (001) TiO_2_ NT (0,40) and its 2D FVS model, with a multiplied unit cell.

The section of results for the (001) structures also begins with a discussion on geometry. In Figure 10 and Figure 11 fragments of (001) NTs (40,0) and (0,40) with water are shown, together with their 2D FVS models. It is possible to see the similarity between the corresponding structures, although there is no curvature in the 2D models. In the case of the (001) NTs, also water orientation agreement is better. It is visible that not only the water oxygen but also both hydrogen atoms have similar positions in both (*n*,0) and (0,*n*) cases. The general trend is that the 2D structure keeps adequate similarity to the reference NT down to chirality indices of 20–16.

**Figure 12:**
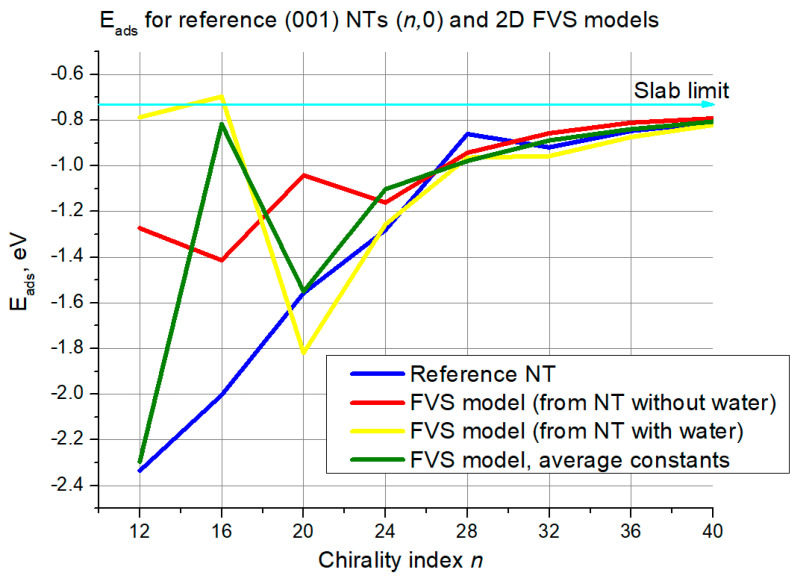
Adsorption energies for (001) NTs (*n*,0) and their 2D FVS models. The cyan line with the arrow denotes the slab limit for water adsorption energy.

Water adsorption energies are shown for (001) (*n*,0) structures in Figure 12. Again, there is a curve for the reference NTs, a curve for each of the three FVS model variations, and the regular slab limit is also marked, which is −0.72 eV in the (001) case.

As is seen in Figure 12, the reference curve grows rather monotonically, with a single exception of the peak at *n* = 28, and converges to the slab limit. In turn, the FVS model curves exhibit chaotic behavior in the interval of lower chirality indices (12–20), sometimes approaching the reference curve accidentally. Solid agreement between the reference and the FVS model curves begins at about *n* = 20–24. There, the maximum deviation is lower than 0.4 eV, and it becomes negligible moving forward to higher *n* values. It is difficult to distinguish the best-performing FVS model variation until *n* = 32. However, when *n* > 32 the FVS model variation based on averaged constants performs better than the other two.

**Figure 13:**
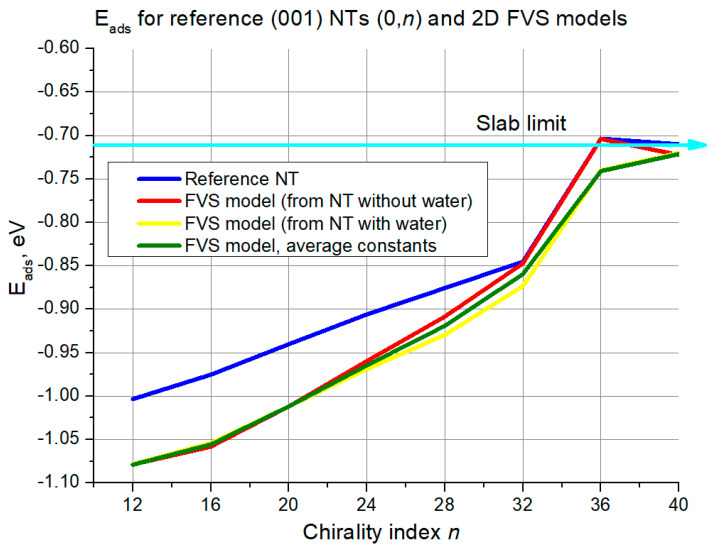
Adsorption energies for (001) NTs (0,*n*) and their 2D FVS models. The cyan line with the arrow denotes the slab limit for water adsorption energy.

Figure 13 shows that the reference curve growth monotonically in the interval *n* = 12–32, then there is a jump between 32–36 and a plateau-like interval of 36–40. There is no chaotic curve behavior in the domain of low chirality indices. Still, the FVS curves all start with an offset of around 0.075 eV with respect to the reference curve. In the interval *n* = 12 –32, this offset gradually diminishes until *n* = 32, where the curve for FVS models based on NTs with water overlaps with the reference curve, and then diverges somewhat between *n* = 36 and 40. For the other two slab model variations the offset grows to 0.05 eV at *n* = 36, and then the curves begin to approach the reference again.

**Figure 14:**
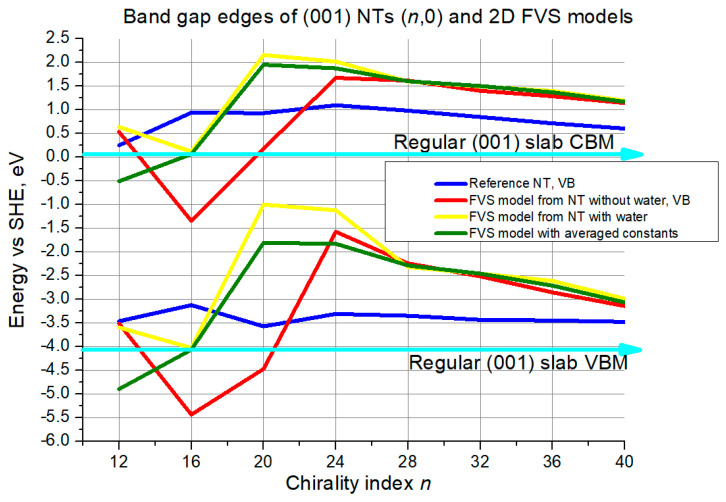
VB top and CB bottom edge positions for (001) NTs (*n*,0) and their 2D FVS models. The cyan lines with arrows denote slab limits for VB maximum and CB minimum.

**Figure 15:**
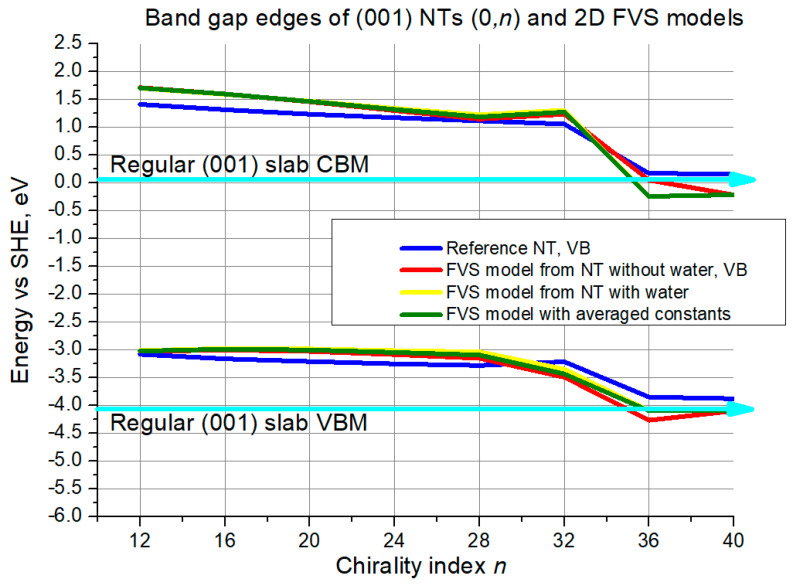
VB top and CB bottom edge positions for (001) NTs (0,*n*) and their 2D FVS models. The cyan lines with arrows denote slab limits for VB maximum and CB minimum.

Next, the criterion of band edge positions will be discussed based on Figure 14 and Figure 15.

The slab limits are slightly above 0 eV and below −4.0 eV for CBM and VBM, respectively. The curve for the reference NTs does not fluctuate essentially, 0.5 eV at most for the CBM and even less for the VBM, returning close to the starting level when reaching chirality index 40. The 2D model curves exhibit chaotic behavior in the range of smaller chirality indices, as it has already been observed for the (*n*,0) structures. These curves get close to each other at *n* = 24–28 and further on. The deviation is large, though, around 0.5 eV for the CBM along the whole interval *n* = 24–40, but for the VBM the deviation decreases from around 1.0 eV at *n* = 28 to around 0.5 eV at *n* = 40.

The situation is quite different for the (001) (0,*n*) structures. The reference curves descend monotonically from the beginning until *n* = 32, then there is a steeper descent until *n* = 36, and a plateau until *n* = 40. The FVS model curves do not exhibit chaotic behavior in any range. For the CBM they begin with an offset of around 0.4 eV with respect to the reference, which then decreases to almost 0 until *n* = 28, and then increases again. The red curve remains the closest to the reference until *n* = 36, but it diverges later. The two other curves reproduce the same plateau in the interval *n* = 36–40.

For the VBM all the curves start from the same point at *n* = 12. Then the reference curve and 2D model curves follow slightly different patterns until *n* = 32, with the deviation reaching 0.25 eV at most. Then the patterns become similar. The deviation reaches 0.5 eV again for the curve of FVS models based on NTs without water, and stays at around 0.25 eV for the other slab model curves. Though, the red curve approaches the reference in the interval of *n* = 36–40.

## 4. Summary and Conclusions

With the results discussed above, we ensured additional validation of the 2D FVS model which was developed by us earlier for facilitation of ab initio MD simulations of water-covered NTs. In this study, the novelty in the model validation was different NT configurations as material for testing of the single model. For each of the configurations, the model performance was tested both in the domain of small NT diameters where the strain effects are high and in the interval of higher chirality indices where NT properties already approach those of a slab.

The first validation criterion was water adsorption energy. In the case of (101) NTs, the FVS model demonstrated solid reproduction of the reference data, with the largest deviation of 0.15 eV. The most accurate model in this case (the variation with averaged constants) exhibited negligible deviations at the higher chirality indices. In turn, for the (001) NTs the largest deviation was even higher than 1.0 eV for the (*n*,0) configuration, while for the (0,*n*) configuration it was always lower than 0.1 eV, i.e., negligible in the whole range. Still, even for the (*n*,0) configuration, the difference decreases and also becomes negligible starting from chirality indices of around 24.

The second validation criterion was positions of band edges. For the (101) structures the agreement between the reference NTs and their 2D models was satisfactory, with deviations ranging between 0.5 and 0.25 eV for the (*n*,0) configuration. For the (0,*n*) configuration the results were better, with maximum deviations around 0.25 eV and with negligible deviations towards medium and large-size NTs. In the case of the (001) (*n*,0) structures the agreement was weaker, especially considering the chaotic behavior, so that acceptable deviation of 0.5 eV for both gap edges simultaneously was reached only at the largest NT diameter. For the (001) (0,*n*) configuration, in turn, the agreement was much better, not exceeding 0.25 eV in the whole interval.

To sum up the results, the 2D FVS model in general performed well for most NTs starting from medium chirality indices, around 20–28, both in terms of water adsorption energy and band edge reproduction. In the case of bandgap edge validation for (001) (*n*,0) structures performance of the slab models was imprecise until the largest diameter. On the contrary, the 2D model performance was good even for lower chirality indices in multiple cases. Geometry reproduction was satisfactory for (101) NTs, and good for (001) NTs. In most cases, the FVS model based on averaged lattice constants was the most accurate, although there were exceptions.

This study has contributed with additional validation to the 2D slab models proposed by us earlier [8,13], which have the potential of facilitating computationally expensive calculations, such as ab initio MD. The novelty of this work consists in validating the same 2D FVS model against 6-layered TiO_2_ NTs with a rectangular unit cell and with different configurations: (101) (*n*,0), (101) (0,*n*), (001) (*n*,0), and (001) (0,*n*), and positive results were obtained for every case. This broadens the conclusions of our previous work to the statement that these models are potentially applicable to NTs of other materials built from rectangular unit cells. A topic for further studies on the construction of reduced computational models could be the performance of 2D models for NTs with hexagonal configuration, although the model construction would be more cumbersome since chirality vectors are often not parallel to the circumference and main axis in a general case of a hexagonal NT.

## Figures and Tables

**Table 1:** Parameters of 6-layered (101) (*n*,0) TiO_2_ NTs, simulated with and without water. The lattice parameter corresponding to NT axis is denoted with *a*, d stands for outer diameter, *b*—for outer *b* constant along NT circumference, and *a* × *b*—their product.

(101) (*n*,0)	NTs with Clean Surface				NTs with Water Adsorbed			
Chirality Index *n*	d Outer	*a*	*b*	*a* × *b*	d Outer	*a*	*b*	*a* × *b*
12	16.636	10.411	4.353	45.319	16.954	10.245	4.436	45.451
16	21.043	10.414	4.130	43.006	21.546	10.249	4.228	43.335
20	25.456	10.422	3.997	41.651	26.052	10.258	4.090	41.956
24	29.884	10.425	3.910	40.762	30.564	10.264	3.999	41.045
28	34.326	10.430	3.849	40.149	35.116	10.265	3.938	40.422
32	38.757	10.435	3.803	39.685	39.666	10.267	3.892	39.962
36	43.237	10.435	3.771	39.352	44.246	10.267	3.859	39.624
40	47.674	10.439	3.742	39.068	48.824	10.269	3.833	39.358

**Table 2:** Parameters of 6-layered (101) (0,*n*) TiO_2_ NTs, simulated with and without water. The lattice parameter corresponding to the NT axis is denoted with *a*, d stands for outer diameter, *b*—for outer *b* constant along NT circumference, and *a* × *b*—their product.

(101) (0,*n*)	NTs with Clean Surface				NTs with Water Adsorbed			
Chirality Index *n*	d Outer	*a*	*b*	*a* × *b*	d Outer	*a*	*b*	*a* × *b*
12	42.288	3.540	11.065	39.168	42.086	3.622	11.013	39.885
16	55.585	3.535	10.908	38.566	54.830	3.615	10.760	38.902
20	68.877	3.533	10.814	38.210	68.421	3.612	10.742	38.800
24	82.148	3.535	10.748	37.994	81.589	3.610	10.675	38.533
28	95.498	3.529	10.709	37.798	94.755	3.609	10.626	38.346
32	108.725	3.534	10.669	37.704	107.924	3.608	10.590	38.206
36	122.095	3.529	10.649	37.580	121.094	3.607	10.562	38.098
40	135.402	3.529	10.629	37.506	134.260	3.606	10.539	38.010

**Table 3:** Parameters of 6-layered (001) (*n*,0) TiO_2_ NTs, simulated with and without water. The lattice parameter corresponding to the NT axis is denoted with *a*, d stands for outer diameter, *b*—for outer *b* constant along NT circumference, and *a* × *b*—their product.

(001) (*n*,0)	NTs with Clean Surface				NTs with Water Adsorbed			
Chirality Index *n*	d Outer	*a*	*b*	*a* × *b*	d Outer	*a*	*b*	*a* × *b*
12	19.809	3.601	5.183	18.664	20.044	3.583	5.245	18.792
16	22.900	3.664	4.494	16.468	24.193	3.573	4.748	16.963
20	25.265	3.743	3.967	14.846	27.264	3.784	4.280	16.195
24	28.448	3.772	3.722	14.039	30.939	3.822	4.048	15.471
28	31.938	3.782	3.582	13.545	32.462	3.695	3.640	13.453
32	35.558	3.785	3.489	13.206	36.502	3.682	3.582	13.189
36	39.164	3.787	3.416	12.935	40.244	3.691	3.510	12.956
40	42.841	3.787	3.363	12.736	43.349	3.732	3.403	12.700

**Table 4:** Parameters of 6-layered (001) (0,*n*) TiO_2_ NTs, simulated with and without water. The lattice parameter corresponding to the NT axis is denoted with *a*, d stands for outer diameter, *b*—for outer *b* constant along NT circumference, and *a* × *b*—their product.

(001) (0,*n*)	NTs with Clean Surface				NTs with Water Adsorbed			
Chirality Index *n*	d Outer	*a*	*b*	*a* × *b*	d Outer	*a*	*b*	*a* × *b*
12	20.628	3.247	5.398	17.528	20.380	3.250	5.333	17.333
16	25.468	3.249	4.998	16.240	25.323	3.259	4.970	16.194
20	30.252	3.250	4.750	15.436	30.229	3.266	4.746	15.499
24	34.972	3.249	4.576	14.868	35.104	3.272	4.593	15.028
28	39.636	3.247	4.445	14.434	39.943	3.280	4.479	14.691
32	44.295	3.246	4.346	14.108	44.774	3.280	4.393	14.410
36	47.684	3.014	4.159	12.533	47.640	3.016	4.155	12.530
40	52.461	3.014	4.118	12.412	52.368	3.017	4.111	12.404

## Data Availability

The raw/processed data required to reproduce these findings cannot be shared at this time as the data also form a part of an ongoing study.
